# Physicians’ Attitudes towards and Reasons for Participation in the Candesartan Antihypertensive Survival Evaluation in Japan (CASE-J) Trial

**DOI:** 10.2188/jea.15.38

**Published:** 2005-05-10

**Authors:** Mahbubur Rahman, Satoshi Morita, Tsuguya Fukui, Junichi Sakamoto

**Affiliations:** 1 Clinical Practice Evaluation and Research Center, St. Luke’s Life Science Institute & St. Luke’s International Hospital.; 2 Department of Epidemiological and Clinical Research Information Management, Kyoto University Graduate School of Medicine.

**Keywords:** Physicians, Randomized Controlled Trials, Data Collection, Information Systems, Database Management Systems

## Abstract

BACKGROUND: Physicians’ perception and attitudes towards a research topic and trial management could influence their participation in a randomized controlled trial. The objectives of this study were to determine the reasons for physicians’ participation in and attitudes towards the Candesartan Antihypertensive Survival Evaluation in Japan (CASE-J) trial.

METHODS: CASE-J’s main objective is to compare the effectiveness of an angiotensin II receptor antagonist (candesartan cilexetil) with that of a calcium channel blocker (amlodipine besilate) in terms of the incidence of cardiovascular events among high-risk hypertensive patients. We conducted a questionnaire survey among the physicians (n=512) participating in that trial to determine the reasons behind their participation and to elicit their reactions to the trial management.

RESULTS: Eighty-eight percent of the 512 participating physicians responded to our survey. The main reasons for participation were clear objectives of the trial (85.1%), a simple protocol (61.1%), interest in finding out the inhibiting effects of the drugs on cardiac events (80.2%), and a well-organized support system (59.8%). As for negative factors, case registration and follow-up were considered cumbersome by 28.6% and 10.8%, respectively while 44.2% stated that support by the clinical research coordinators provided by the trial management authority was necessary for case screening, recruitment process, patient registration, and follow-up. Multivariate logistic regression analysis showed that participants who did not use a computer very regularly (odds ratio = 1.9, 95% confidence interval = 1.1-3.6) were more likely to consider the case registration and follow-up procedures as a cumbersome.

CONCLUSION: The information generated by this study could be useful in designing future randomized controlled trials in Japan and abroad.

A randomized controlled trial (RCT), the most important method for generating high-grade evidence, is essential for progress in medicine. In addition to the rationale of the trial, there are many components of the RCT which should be taken care of for the smooth running of a trial. Easy patient recruitment and randomization, well-designed follow-up procedures, facilitators for physician participation, effective data management, and satisfaction of both physicians and patients with the trial activities are the main pillars of a successful RCT. A recent systematic review identified major barriers to physicians’ participation in RCTs.^1^ However, reasons for participation by Japanese physicians in clinical trials, their attitudes towards them, and facilitators and barriers have not been reported yet.

The Candesartan Antihypertensive Survival Evaluation in Japan (CASE-J) is a prospective, multicenter, open-label, randomized controlled trial for high-risk hypertensive patients to compare the effectiveness of a angiotensin II receptor antagonist (candesartan cilexetil) and of a calcium channel blocker (amlodipine besilate) in terms of the incidence of cardiovascular events.^2^ Enrollment began in September 2001 and follow-up is to be completed in December 2005. It is the first RCT on this scale in Japan using Web-based technology for patient registration, their follow-up, and documentation procedures. Although the patient registration is completed and follow-up has been continuing on schedule, it is extremely important to determine which aspects of the trial appeal to the physicians, which aspects remain problematic, and what needs to be added or changed to make the trial more acceptable to the participating physicians. Thus the objectives of this study were to survey the participating physicians in this regard with the hope that the results would be useful for the clinical trial planners in Japan and abroad.

## METHODS

The parent study has been previously described.^3^ Briefly, the questionnaires were sent to all 512 physicians participating in the CASE-J trial in January 2003, immediately after the patient recruitment period, which ended in December 2002. The part of questionnaire related to the objective of this study comprised eight questions about the reasons for participation in the trial and six about their attitudes towards it. The responses to the questions were rated on a five-point scale (strongly agree, agree, neutral, disagree, and strongly disagree).

To identify the predictors about participation and particular attitudes (dependent variables), we performed multivariate logistic regression analyses in which age, sex, specialty of practice, working place, clinical study courses taken, prior experience with other clinical trials/studies, current participation in other clinical trials, computer use and the number of patients they recruited were considered independent variables. Each of the opinions was considered a dependent variable (agree vs. disagree plus neutral) and separate logistic regression models were used to identify the predictors if any. In this regard, ‘strongly agree’ and ‘agree’, ‘strongly disagree’ and ‘disagree’ were combined into ‘agree’ and ‘disagree’, respectively. STATA^®^ statistical software was used for the statistical analyses.^4^

## RESULTS

A total of 448 physicians (87.5%) returned the questionnaire. Details of demographic characteristics of the respondents have been published elsewhere.^3^ Table 1 shows the factors which made physicians decide to participate in the CASE-J trial. The main reasons were clear objectives (85.1%), a simple protocol (61.1%), interest in finding out the inhibiting effects of the drugs on cardiac events (80.2.%), and a well-organized support system provided by the trial management committee (59.8%). Other less important reasons included interest in Web-based trial management systems (37.3%), recommendation by other physicians involved in the CASE-J trial (32.1%), ease of persuading patients to take part in this trial (21.4%), and their hospital’s decision to participate (15.5%) (Table 1). On the other hand, case registration or follow-up procedures were considered cumbersome by 31.0% of the physicians (28.6% for case registration and 10.8% for follow-up; Table 2). Fifty-four percent of the participants were satisfied with the incentives provided by the trial authority while 44.2% stated that additional support from clinical research coordinators was necessary for case screening, recruitment process, patient registration and follow-up. More than 21% replied that there is room for improvement, specifically for registration (17.5%) and follow-up procedures (9.1%). Multivariate logistic regression analysis showed that participants who did not use a computer very regularly (odds ratio = 1.9, 95% confidence interval = 1.1-3.6) were more likely to consider case registration and follow-up procedures cumbersome. No other factors were found to be predictive of the reasons for physicians’ participation or their attitudes.

**Table 1.** Reasons of physicians’ participating in the Candesartan Antihypertensive Survival Evaluation in Japan (CASE-J) trial. (n=448)

Statement	Physicians’ response (%)		

	Agree	Neutral	Disagree
Agreement with aims of the study.	85.1	13.1	1.8
Simplicity of study protocol.	61.1	24.6	14.2
Special interest in the comparison of the treatments in CASE-J	80.2	16.1	3.7
Special interest in the use of Internet for this trial	37.3	32.8	29.9
Expectation that it would be easy to convince patients to enroll in this trial	21.4	35.0	43.7
Well-organized support system for case registration and follow-up	59.8	30.1	10.2
Recommendation by doctors involved in CASE-J	32.1	27.3	40.6
Decision by hospital where they work to participate in CASE-J	15.5	24.5	60.0

‘Strongly agree’ and ‘agree’, strongly disagree and ‘disagree’ were combined into ‘agree’ and ‘disagree’, respectively.

Figures do not always add up to 100% due to rounding.

**Table 2.** Physicians’ opinions about the Candesartan Antihypertensive Survival Evaluation in Japan (CASE-J) trial. (n=448)

Statement	Physicians’ response (%)		

	Agree	Neutral	Disagree
Procedure for case registration is cumbersome.	28.6	28.6	42.7
Procedure for case registration should be improved.	17.5	37.5	45.0
Procedure for follow-up study is cumbersome.	10.8	36.8	52.4
Procedure for follow-up study should be improved.	9.1	40.6	50.4
Compensation for case registration or follow-up is appropriate.	54.3	38.8	6.9
CRC support should be provided for screening and qualification of cases, patient registration, and follow-up study.	44.2	37.5	18.3

CRC: clinical research coordinators

‘Strongly agree’ and ‘agree’, strongly disagree and ‘disagree’ were combined into ‘agree’ and ‘disagree’, respectively.

Figures do not always add up to 100% due to rounding.

## DISCUSSION

This survey demonstrated that clear objectives, simple protocol, scientific validity of the trial, and a well-designed support system were the main reasons behind physicians’ participation while 31% of them mentioned trouble with either case registration or follow-up procedures, and 21% considered that there is room for improvement of this system. The results of this study indicated that, although the CASE-J trial has been using highly sophisticated technology (Web-based data entry system), there is a need for more efficient trial management procedures according to the participating physicians. A more detailed survey about the problems they have been facing and their suggestions for improvement should be helpful for future trials in Japan and abroad.

This study also showed that physicians who were not very regular computer users were more likely to report problems with registration and follow-up. Not surprisingly, this group of physicians also stated that they did not feel comfortable with Web-based data entry procedures. The following measures could be useful for overcoming the problems related to data entry for case recruitment and follow-up procedures. First, recruitment of clinical research coordinators or other research staff to assist the participating physicians is essential. Although CASE-J has clinical research coordinators to assist with these problems, the extent of cooperation needed should be determined by surveying the participating physicians at the beginning of any such study, because the needs may vary depending on the trials and physicians. Second, a system should be established in which trial-related documents could be faxed to the central coordinating unit, where data entry would be performed for those who prefer not to do this by themselves. This system is already present in the CASE-J trial. Third, a demonstration, along with an intensive teaching session, of the Web-based system for the physicians, preferably in person, is a prerequisite for successful implementation of this type of study.

In this study we found that clear objectives, a simple protocol, and rational hypotheses are the main factors which attract physicians to participate in an RCT. A sizeable number of physicians had trouble with case registration and follow-up procedures, although the system is considered highly suitable for conducting an RCT. Appropriate corrective measures should be taken for future trials in Japan by taking physicians’ preference and satisfaction into account.
